# The degree of risk factor and accumulation effect for large niche in individuals after cesarean section

**DOI:** 10.1186/s12884-023-06228-7

**Published:** 2024-01-05

**Authors:** Jing Wang, Ye He, Mengyuan Zhang, Fen Huang, Yuanyuan Wu, Mingjun Hu, Yuanyuan Yang, Wenwen Wei, Qiushi Pang, Zhaolian Wei

**Affiliations:** 1 Department of Obstetrics & Gynecology, First Affiliated Hospital of Anhui Medical University, Hefei, 230020 Anhui People’s Republic of China; 2 Anhui Province Key Laboratory of Reproductive Health and Genetics, Hefei, 230020 Anhui People’s Republic of China; 3 Department of Epidemiology and Biostatistics, School of Public Health, Anhui Medical University, Hefei, 230032 Anhui People’s Republic of China; 4 Department of Ultrasound, First Affiliated Hospital of Anhui Medical University, Hefei, 230032 Anhui People’s Republic of China; 5 Department of Obstetrics & Gynecology, Fourth Affiliated Hospital of Nanjing Medical University, Nanjing, 210031 Jiangsu People’s Republic of China; 6 Shanghai Key Laboratory of Maternal Fetal Medicine, Department of Fetal Medicine & Prenatal Diagnosis Center, Shanghai First Maternity and Infant Hospital, School of Medicine, Tongji University, Shanghai, 200092 People’s Republic of China

**Keywords:** Cesarean section, Large niche, Risk factor, Accumulation effect

## Abstract

**Background:**

The risk factors associated with niche on the cesarean scar have been reported, however, the degree of these factors associated with large niche and the accumulation effects of these risk factors on the development of large niche are unclear.

**Methods:**

Large niche was evaluated by transvaginal sonography during mid-follicular phase. Logistic regression model was used to assess 32 risk factors by univariate analysis. Then, a scoring model based on the screened risk factors was generated. The performance of this model was evaluated by area under curve (AUC). Finally, the scoring model was applied in 123 women to assess the external validation.

**Result(s):**

In the training cohort study, 163 women were diagnosed with large niche. The final scoring model involves eight risk factors with the rating scores including age at delivery (30–34 years: 1 point; ≥ 35 years: 4.5 points), retroflexed uterus (8.5 points), meconium-stained amniotic fluid (4.5 points), twice CSs (4.0 points), postpartum endometritis (4.5 points), premature rupture of membranes (2.5 points), intrahepatic cholestasis of pregnancy (mild to moderate: 3 points; severe: 6.5 points), and cervical dilatation (1-3 cm: 2.0 points; 4-10 cm: 4.5 points). The accumulation effect with a cut-off value of 8.0 in the scoring was associated with the large niche after CS.

**Conclusion(s):**

This is the first scoring model to objectively quantify the risk of a large niche after CS. Optimal risk factors control by avoiding high score factors and multiple factors accumulation may eliminate the risk of large niche development.

**Supplementary Information:**

The online version contains supplementary material available at 10.1186/s12884-023-06228-7.

## Background

Parallel to the rise of cesarean section (CS) rates, the incidence of its relevant uterine niche and niche complications have also been increased dramatically. The complications of the large niche after CS were of the most consideration. Compared with the small niche, the large niche is more likely to impair uterine strength and to be associated with severe postmenstrual spotting and uterine rupture in the subsequent labor.The prevalence of large niche variates from 11.0% to 28.3% because of no consistent definition [1, 2]. Our previous study has objectively defined the large niche according to depth, residual myometrial thickness (RMT) and adjacent myometrial thickness (AMT) based on postmenstrual spotting in a large sample [2], and according to our definition the prevalence of large niche was 22.4% [2].

Although the factors associated with niche formation have been widely reported, the degree of these risk factors specifically associated with the development of large niche remain unclear and no validated scoring system has been developed to screen a cut-point value for large niche to achieve more efficient risk control. In general, the factors related to the size of a niche could be classified into four categories including those related to closure technique, development of the lower uterine segment or location of the incision, wound healing and miscellaneous factors [3, 4]. Among them, risk factors of large niche might be single-layer myometrium closure, multiple CSs and retroflexed uterus, but the findings were controversial and limited to the small sample size. Single-layer closure has been suggested to be associated with higher prevalence of large niches compared with double-layer closure [4–6], however, a recent multi-center, double-blind, randomized controlled superiority trial concluded there was no significant different between single and double-layer regard to the formation of large niche [7]. Several studies have reported that multiple CSs was associated with large niche [1, 8, 9] but Monteagudo et al. [10] did not find any association between the number of CS and defect size. Above all, the development of large niche should be considered as the results of the coefficient of multiple factors but not a single risk factor, and each factor may contribute variously. Therefore, a risk scoring model with multiple factors for large niche is needed to provide an overall evaluation system for calculating risk.

Hence, this study aimed to develop a score-based model with multiple risk factors in a relatively large sample size to quantify the single risk factor and the accumulation risk for the development of large niche.

## Methods

### Study population

The current study was the second part of our cohort study on large niche and the first part on the definition of large niche has been published previously [2]. The participants of this study were a subgroup of women from the original cohort and the study design has been described previously [2]. The study protocol was approved by the Ethics Committee of the First Affiliated Hospital of Anhui Medical University, China (PJ2019-03–12). All the participants had filled out an informed consent before participating in the study.

The whole cohort study retrospectively included 2,141 non-pregnant women who had their last CS for more than one year between January 2012 to February 2021 in First Affiliated Hospital of Anhui Medical University. A total of 850 women received the transvaginal sonography (TVS) examination by 2D color Doppler All the participants were invited to complete questionnaires, including questions on menstruation cycle, methods of contraceptive, dysmenorrhea, abnormal uterine bleeding, infertility, dyspareunia, gynecological endocrine disease, whether had another baby or underwent other surgeries. Clinical information on blood testing, body temperature before and after CS, pregnancy history, indications of operation and delivery were obtained from the electronic medical database. Women with one or twice CSs history were included in our study, as the size of sample with more than twice CSs was very small.

**Fig. 1:**
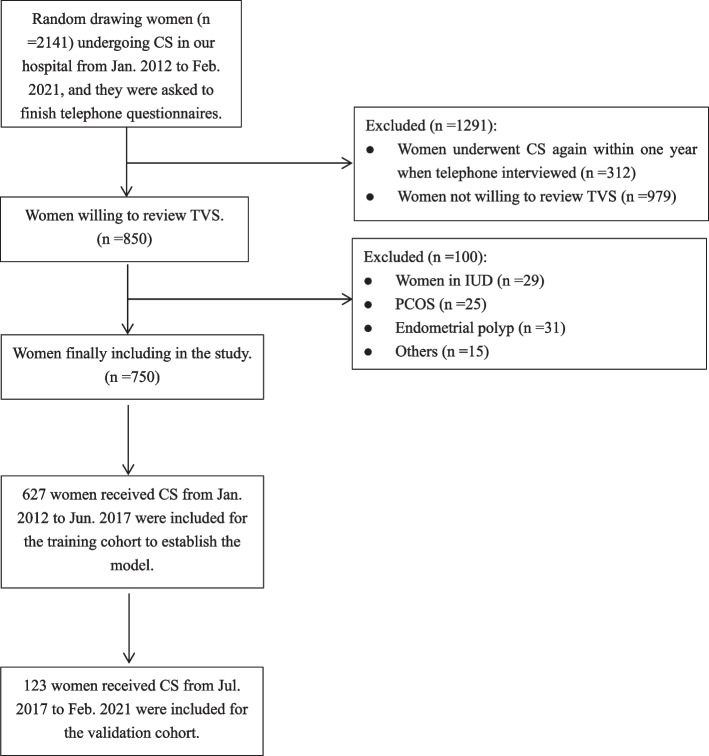
Flow chart of patients included in the study

Finally, a total of 750 women participant this study (Fig. 1). The scoring model was developed based on the training cohort study of 627 women. The model was tested on an independent validation cohort study of 123 women.

### Cesarean scars measurement

Delineation and measurements of the CS scar were conducted according to the methods described in our previous study [2]. Briefly, the uterus position, endometrium thickness, residual myometrial thickness (RMT), adjacent myometrial thickness (AMT) of the scar, RMT (i.e., for the complex niche, recording the thinnest RMT), depth, length, and width were measured for the niche [11, 12] during the mid-follicular phase of menstruation. All values were taken as the average of three examinations. Large niche was defined as more than 0.50 cm in depth, or less than 0.21 cm in RMT, or more than 0.56 in depth/AMT [2].

### Risk factor

A total of 31 variables related to large niche were identified based on the review, meta-analyses, and high-quality studies [3, 13]. They were divided into four categories, including operation, inflammation or infection, tension, and healing related (Table S1). It should be emphasized that the duration of premature rupture of membranes (PROM) here is more than 24 h before CS. The definition of postpartum endometritis is oral temperature ≥ 38.0℃ any two of the first 10 days postpartum, or ≥ 38.7℃ during the first 24 h postpartum. The other criteria of risk factors were shown in SI.

Although, the studies from JAF Huirne showed double-layer closure with unlocked sutures and exclusion of the decidual is advantageous of CS incision healing, the method of wound closure (single vs. double layer sutures) was not employed as a risk factor [5, 7, 14, 15] in the current study because a continuous locked single-layer uterine suture with peritoneal closure and inclusion of the decidua was applied to all in our department.

### Statistical analysis

#### Univariate analyses and multivariate logistic regression analyses of risk factors of a large niche

Univariate analyses between two groups were conducted using the *t-*test for continuous variables, and the *chi-squared* test was carried out for categorical variables. The variables with *p* < 0.20 in univariate analyses were included in the multivariate logistic regression analysis. The odds ratio (OR) and 95% CI (confidence interval) for the association of a large niche with predictors were estimated using logistic regression, and a *p* < 0.05 was considered to be statistically significant.

#### Scoring prediction model for quantifying risk of the large niche formation

The risk scores of each predictor in the model were calculated by dividing the minimum *β*-coefficient from the logistic regression and rounding to the nearest 0.5. The minimum *β*-coefficient for each predictor was default assigned as one score. The total risk score of each participant was calculated by summing the scores of each predictor, and then, a score-based model of risk factor was developed.

#### The cut-off points for the accumulation effect on large niche

The discriminative ability of the models was evaluated by AUC and its 95% CI, and the goodness of fit of this model was assessed by Hosmer–Lemeshow test. We additionally calculated the statistics of the model performance using the leave-one-out and cross-validation strategies to assess the generalization ability of this prediction model. The formulas of the predictive model were show as below.

Logit (P) = -2.984 + 0.282 * Age at delivery (30 y—34 y) + 1.220 * Age at delivery (≥ 35 y) + 1.093 * Twice CSs + 0.643 * PROM + 1.328 * MSAF + 0.499 * Cervical dilatation (1 cm—3 cm) + 1.288 * Cervical dilatation (4 cm—10 cm) + 2.451 * Retroflexed uterine + 1.205 * Postpartum endometritis + 0.785 * Mild to moderate ICP + 1.864 * Severe ICP.

The 95% IC of specificity and sensitivity were calculated according to the formulas below.

$$\begin{array}{cc}{S}_{p}=\sqrt{\frac{{S}_{p}\left(1-{S}_{p}\right)}{c+d}}& \left({\text{CI}}:{S}_{p}-{{\text{z}}}_{\alpha /2}{S}_{p},{S}_{p}+{{\text{z}}}_{\alpha /2}{S}_{p}\right)\end{array}$$

$$\begin{array}{cc}{S}_{p}=\sqrt{\frac{{S}_{e}\left(1-{S}_{e}\right)}{a+b}}& \left({\text{CI}}:{S}_{e}-{{\text{z}}}_{\alpha /2}{S}_{p},{S}_{e}+{{\text{z}}}_{\alpha /2}{S}_{p}\right)\end{array}$$

In the validation cohort, kappa coefficient (κ) was used to determine agreement between the new definition and the score-based prediction model for large niches. For kappa coefficient, it is generally considered that 0.4 < kappa < 0.6 indicates general consistency, 0.6 < kappa < 0.8 indicates high consistency, kappa > 0.8 indicates good consistency, and kappa < 0.4 indicates poor consistency.

### Sample size analysis

We predicted the sensitivity of the new definition of large niche was 70%, and the specificity for the new definition was 80% according to the previous large definition. The sample size was calculated as the follow formula.

$$n=\left(\frac{{Z}_{\alpha }}{\delta }\right)2{\text{p}}\left(\alpha =0.05, \delta =0.05\right)$$

The sample size should be at least 126 patients for the large niche group, and 96 patients for the control group. In the current study we have included 163 women for the large niche group and 464 women for the control group.

All statistical analyses were performed using the SPSS 23.0 (IBM Corp., Armonk, New York). The data were presented as mean ± SD for normally distributed variables and frequency (percentage, %) for categorical variables.

## Results

In the training cohort study (*n* = 627), 163 women diagnosed with large niche were classified into the large niche group, and 464 women without visible niche under the ultrasound were classified into the control group. In the validation cohort (*n* = 123), there were 40 women with large niche, and 83 women without large niche.

### Demographic characteristics

The basic characteristics of the participants were shown in Table S2. The prevalence of a large niche was 27.1% (203/750) according to our definition. The participants were aged between 22 to 44 years, with a mean age of 29.87 ± 3.84 years, and gestational age ranged from 30 to 42 weeks at CS, with a mean value of 38.62 ± 1.94 weeks. Among these women, 37 had a vaginal delivery, 143 had twice CSs, and 307 had an abortion (ranged from 1 to 9 times). The parity of participants ranged from 1 to 3, with a mean of 1.14 ± 0.59.

### Risk factors related to the formation of a large niche

Table S1 shows the results of univariate analyses of the candidate predictive variables of a large niche. As shown in the table, women with a large niche were inclined to be multiparous, more than 35-years old at delivery, twice CSs, bilateral tubal ligation, emergency CS, postpartum endometritis, meconium-stained amniotic fluid (MSAF), cervical dilatation (4–10 cm), premature rupture of membranes (PROM), multiple vaginal examinations during labor, retroflexed uterus, presence of labor before CS, duration of labor before CS, oxytocin augmentation during labor, and intrahepatic cholestasis of pregnancy (ICP).

### Multivariate logistic model and assigned scores

Table 1 shows the risk factors associated with a large niche in the multivariable logistic model and the assigned scores. The final model includes the following nine risk factors: age at delivery (0 for ≤ 29 years, 1.0 for 30—34 years, 4.5 for ≥ 35 years), twice CSs (4.0 for yes), MSAF (4.5 for yes), PROM (> 24 h, 2.5 for yes), cervical dilatation (0 for 0 cm; 2.0 for 1—3 cm; 4.5 for 4—10 cm), ICP (3.0 for mild, 6.5 for severe), postpartum endometritis (4.5 for yes) and retroflexed uterus (8.5 for yes).

**Table 1:** Risk factors associated with large niche in the multivariable logistic model and the assigned scores

	Regression coefficient	Adjusted OR (95% CI)	Assigned Scores
Age at delivery (y)			
≤ 29		1.00 (reference)	0
30—34	0.282	1.326(0.807–2.179)	1
≥ 35	1.220	3.387(1.663–6.897)	4.5
Twice CSs			
No		1.00 (reference)	0
Yes	1.093	2.983(1.678–5.306)	4
PROM (> 24h)			
No		1.00 (reference)	0
Yes	0.643	1.903(1.089–3.323)	2.5
MSAF			
No		1.00 (reference)	0
Yes	1.328	3.773(1.558–9.133)	4.5
Cervical dilatation (cm)			
0		1.00 (reference)	0
1–3	0.499	1.647(0.758–3.579)	2
4–10	1.288	3.626(1.615–8.141)	4.5
Retroflexed uterus			
No		1.00 (reference)	
Yes	2.451	11.605(7.271–18.525)	8.5
Postpartum endometritis			
No		1.00 (reference)	0
Yes	1.205	3.337(1.166–9.549)	4.5
ICP			
No		1.00 (reference)	0
Mild to moderate	0.785	2.191(1.028–4.674)	3
Severe	1.864	6.448(1.547–26.875)	6.5

*CS* cesarean section, *PROM* premature rupture of membranes, *MSAF* meconium-stained amniotic fluid, *ICP* intrahepatic cholestasis of pregnancy

### The cut-off points for the accumulation effect on large niche

**Fig. 2:**
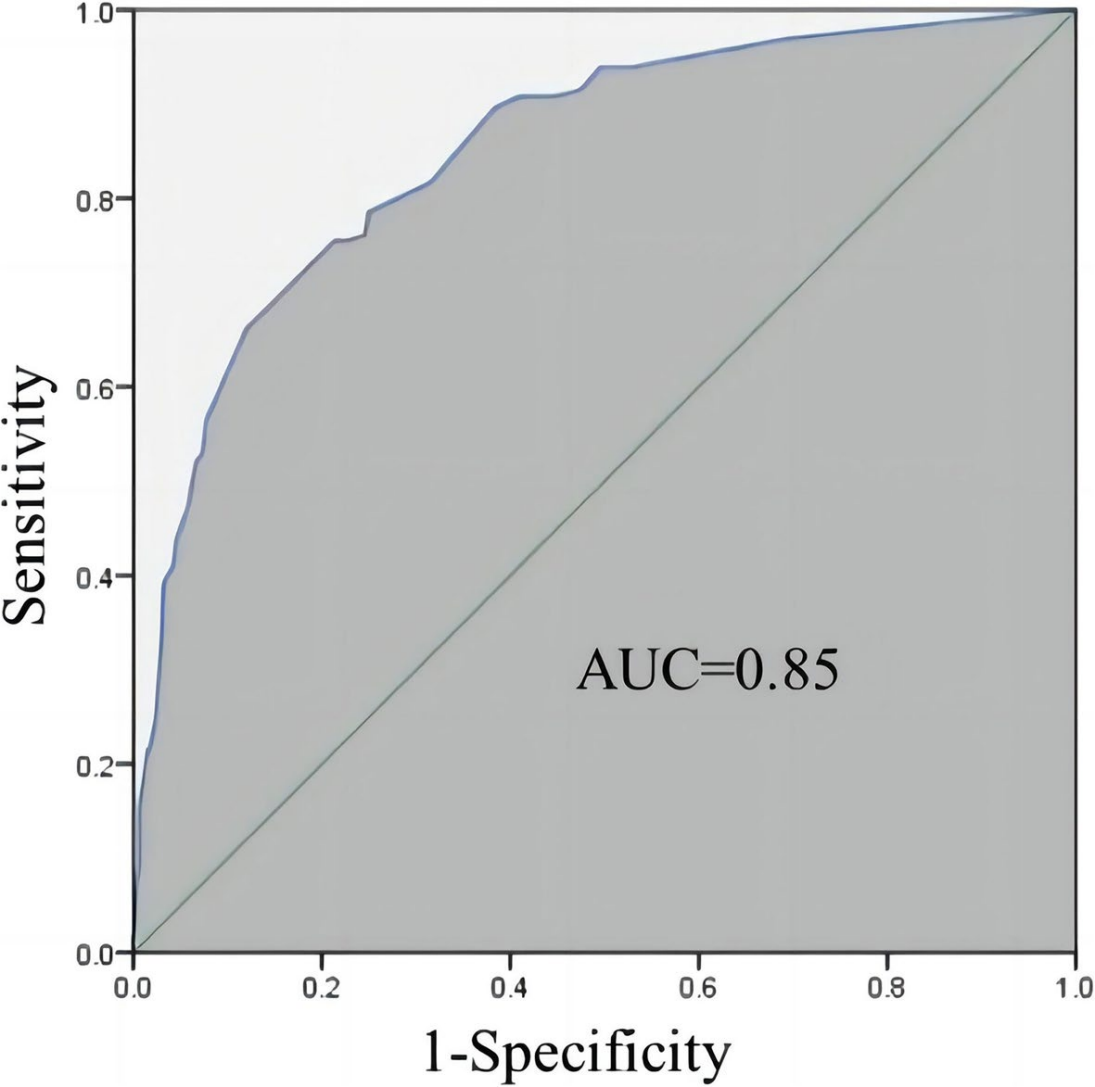
ROC curves for scoring model for prediction of a large niche

Table 2 shows the discriminative performances of each score as the cut-off points in identifying individuals at high-risk of large niche formation in our study. As the cut-off points increased, the risk of large niche formation increased accordingly. We comprehensively estimated some predominant indices for each score cut-off points in the score-based model, including sensitivity, specificity, Youden’s index (sensitivity + specificity – 1), accuracy rate. The overfitting of the prediction model was discussed. Hosmer and Lemeshow test provided Chi-square 10.15 (*p* = 0.180). The ROC curve of this model is shown in Fig. 2. The AUC of the model was 0.85 (95% CI: 0.82–0.89). The leave-one-out cross-validation accuracy of our prediction model is 77.25%.

**Table 2:** Performance of a risk factor scoring model for large niche with different score cut-offs in the study cohort

Score Cut-off	High risk individuals (n, %)	True large niche (n)	Sensitivity (%)	Specificity (%)	Youden's index	Accuracy rate (%)
0	627	163	100.00%	0.00%	0.00	0.00%
1	480	158	96.93%	30.60%	0.28	23.44%
2	398	153	93.87%	47.20%	0.41	36.52%
2.5	383	153	93.87%	50.43%	0.44	38.92%
3	369	149	91.41%	52.59%	0.44	41.15%
3.5	355	148	90.80%	55.39%	0.46	43.38%
4	338	148	90.80%	59.05%	0.50	46.09%
4.5	324	146	89.57%	61.64%	0.51	48.33%
5	279	133	81.60%	68.53%	0.50	55.50%
5.5	251	129	79.14%	73.71%	0.53	59.97%
6.5	244	128	78.53%	75.00%	0.54	61.08%
7	238	124	76.07%	75.43%	0.52	62.04%
7.5	228	123	75.46%	77.37%	0.53	63.64%
8	224	123	75.46%	78.23%	0.54	64.27%
8.5	222	123	75.46%	78.66%	0.54	64.59%
9	172	110	67.48%	86.64%	0.54	72.57%
9.5	164	108	66.26%	87.93%	0.54	73.84%
10	128	92	56.44%	92.24%	0.49	79.59%
10.5	120	86	52.76%	92.67%	0.45	80.86%
11	116	85	52.15%	93.32%	0.45	81.50%
11.5	104	77	47.24%	94.18%	0.41	83.41%
12	92	71	43.56%	95.47%	0.39	85.33%
12.5	87	67	41.10%	95.69%	0.37	86.12%
13	79	64	39.26%	96.77%	0.36	87.40%
13.5	68	54	33.13%	96.98%	0.30	89.15%
14	51	40	24.54%	97.63%	0.22	91.87%
14.5	43	35	21.47%	98.28%	0.20	93.14%
15	42	35	21.47%	98.49%	0.20	93.30%
15.5	38	32	19.63%	98.71%	0.18	93.94%
16	28	25	15.34%	99.35%	0.15	95.53%
16.5	25	22	13.50%	99.35%	0.13	96.01%
17	22	19	11.66%	99.35%	0.11	96.49%
17.5	18	15	9.20%	99.57%	0.09	97.13%
18	14	12	7.36%	99.57%	0.07	97.77%
18.5	13	11	6.75%	99.57%	0.06	97.93%
19.5	9	8	4.91%	99.78%	0.05	98.56%
20	6	5	3.07%	99.78%	0.03	99.04%
21.5	2	2	1.23%	100.00%	0.01	99.68%
31.5	1	1	0.61%	100.00%	0.01	99.84%

### Validation of the scoring model

In the validation cohort (*n* = 123), 49 women were classified into high-risk group, and 34 women diagnosed with large niche. And 74 women were classified into low-risk group, and 6 of them diagnosed with large niche as shown in Table 3. The Kappa value was highest when use the cut-off of 8 and it was 0.63, the sensitivity was 85.00% (95% CI: 73.93%-96.07%), and the specificity was 81.93% (95% CI: 73.65%-90.21%).

**Table 3:** Validation of the scoring model for large niche

	Large niche (%)	postmenstrual spotting
High risk, n (%)	34(69.4)	36(73.5)
Low risk, n (%)	6(8.1)	12(16.2)
*p*	0.000	0.000
Kappa	0.632	0.575

## Discussion

The current study is the first to establish a scoring model to quantify the single risk factor effect and the accumulation effect on the development a large niche after CS. The study has considered a wide range of potential risk factors comprehensively and a final nine variables included in the model with a rating score: age at delivery (0 for ≤ 29 years, 1.0 for 30—34 years, 4.5 for ≥ 35 years), twice CSs (4.0 for yes), MSAF (4.5 for yes), PROM (> 24 h, 2.5 for yes), cervical dilatation (0 for 0 cm; 2.0 for 1—3 cm; 4.5 for 4—10 cm), ICP (3.0 for mild, 6.5 for severe), postpartum endometritis (4.5 for yes) and retroflexed uterus (8.5 for yes). The cutoff points for the accumulation effect on large niche development was 8.0.

Recently, we have proposed a new definition for large niche based on dot bleeding symptom which was more sensitive compared to the previous ones [2]. On the basis of this definition, we further compared the effects of 31 potential risk factors on a large niche after CS. To the best of our knowledge, no score model has quantified the risk factors of a large niche yet. Although several studies have reported the factors that affect the development of large niche [16–18], their single effect and accumulation effect on the contribution to the development of large niche are unknown. Our findings are in agreement with these reported in the literature that age at delivery ≥ 35y, cervical dilation ≥ 4 cm, twice CSs, PROM, and retroflexed uterus are associated with a large niche [16–18]. We have further detailed the degree of each risk factor with an objective rating score and found that the top three risk factors were retroflexed uterine (8.5 points), severe ICP (6.5 points) and MSAF (4.5 points). We also firstly report that PROM more than 24 h should be considered for the large niche. These indicate that hypoxia of the uterus and the resultant inflammatory reaction of uterine incision that may associate with the incomplete healing of CS [19].

The findings of this study also first emphasized that accumulated risk factors effect would lead to the development of a large niche and the cut-off points of 8.0 was determined in the current scoring model. Therefore, the development of large niche could be prevented by avoiding the concurrence of the multiple risk factors. Also, the treatment of obstetric complications actively to reduce the severity would decrease the risk of large niche.

The main strength of this study is that a scoring model for quantification the risk factors the formation of a large niche after CS was proposed for the first time. Second, the sample size were relatively large and risk factors were considered comprehensively which made the model robust and the findings convincible. Third, the measurement of the niche was in mid-follicular period which was consistent with the guideline in European practices [20]. Fourth, the risk factors considered in the model were selected from high-quality literature reviews, meta-analyses, and the consensus from Chinese experts and clinicians. Furthermore, some unknown variables such as MSAF were suggested to be associated with the occurrence of large niche. Finally, the current scoring model had a good fitting degree and proved good discrimination.

Our study also has some limitations. First, a large number of women interviewed (*n* = 979) refused to attend the study, affecting the data collection for developing the scoring prediction model. Second, multiple CSs (i.e., equal or more than three times a history of CS here) was not analyzed in this study, as only few women receiving CS three times in this study. Third, at present, the proposed cut-off points of 8.0 for the large niche scoring model remains to be verified in the clinic to clarify the meaning of guidance for treatment opinions.

## Conclusions

For clinical practice, we have developed a scoring model including eight predictors to quantify the single effect and the accumulation effect on the development of a large niche after CS. It will help clinicians tailor the optimal risk factor control to eliminate the risk of large niche, as well as provide insight into the etiology of large niche.

### Supplementary Information

**Additional file 1:** **Table S1.** Demographic background data and univariate logistic regression analysis. **Table S2.** Demographic characteristics of participants including training and validation cohort.

## Data Availability

The datasets used and analyzed during this study are available from the corresponding author upon reasonable request.

## Abbreviations

CS: cesarean section
RM: Tresidual myometrial thickness
AM: Tadjacent myometrial thickness
TV: Stransvaginal sonography
PROM: Premature rupture of membranes
MSAF: Meconium-stained amniotic fluid
ICP: Intrahepatic cholestasis of pregnancy
OR: Odds ratio
CI: Confidence interval
