# Supplementary material for: The degree of risk factor and accumulation effect for large niche in individuals after cesarean section
Source: BMC Pregnancy Childbirth. 2024 Jan 5;24:38. doi: 10.1186/s12884-023-06228-7 (PMC10768304; doi:10.1186/s12884-023-06228-7)
Supplement: Supplementary file 1 — Additional file 1: Table S1. Demographic background data and univariate logistic regression analysis. Table S2. Demographic characteristics of participants including training and validation cohort. [file 12884_2023_6228_MOESM1_ESM.docx]

The degree of risk factor and accumulation effect for large niche in individuals after cesarean section

Supplementary information

Explanation of variables in univariate logistic regression analysis

Operation: twice CSs, bilateral tubal ligation (i.e., for sterilization), emergency CS, and surgeon experience (i.e., work experience ≥ 10-year).

Inflammation or Infection: meconium-stained amniotic fluid (MSAF, i.e., viscous green material found in the amniotic fluid), cervical dilatation, premature rupture of membranes (PROM, i.e., rupture of membranes before the onset of labor. It should be emphasized that the duration of PROM here is more than 24 hours before CS), and the number of vaginal examinations (i.e., in order to understand the degree of cervical dilatation and presentation engagement). postpartum endometritis (i.e., oral temperature ≥38.0℃ any two of the first 10 days postpartum or ≥38.7℃ during the first 24 hours postpartum)^1, 2^.

Tension: pre-pregnancy bass mass index (BMI), BMI at delivery, retroflexed uterus, macrosomia (i.e., neonatal weight ≥ 4000 g), twin pregnancy, breech, presence of labor, duration of labor (i.e., number of hours with regular contractions), and oxytocin augmentation during labor.

Healing: Pre-eclampsia (PE) was classified into mild and severe (i.e., mild PE is defined as systolic blood pressure at ≥140 mmHg, <160 mmHg, and/or diastolic blood pressure at ≥90 mmHg, <110 mmHg, combined with proteinuria. Severe PE is defined as systolic blood pressure ≥ 160 mmHg, or diastolic pressure ≥ 110 mmHg, combined with proteinuria)^3^, diabetes (i.e., including type I and gestational diabetes, GDM well controlled with diet and exercise was defined as mild group, poorly controlled GDM or type I diabetes required insulin for controlling blood glucose levels were defined as severe)^4^, intrahepatic cholestasis of pregnancy (ICP, i.e., mild was defined as serum total bile aci ≥ 10 μmol/L, ≤ 40 μmol/L, and severe was defined as >40 μmol/L)^5^, anemia (Hb ≤ 90 g/mL), postpartum hemorrhage (PPH, i.e., the amount of bleeding ≥ 1000 mL within 24 hours after delivery), placenta previa (i.e., the placenta completely covers the cervical internal os), steroid treatment during pregnancy (i.e., using estrogen or progesterone during pregnancy) and assisted reproduction technology (ART).

Table S1 Demographic background data and univariate logistic regression analysis

|  | Control group  (n =464) | Large niche group  (n =163) | *p* | OR |
| --- | --- | --- | --- | --- |
| **Basic information** |  |  |  |  |
| Endometrium thickness, mean ± SD, cm | 0.70 ± 0.23 | 0.71 ± 0.22 | 0.58 | 1.25 (0.57-2.73) |
| Days of menstruation, mean ± SD, d | 6.14 ± 1.63 | 8.80 ± 2.37 | 0.00 | 2.12 (1.85-2.43) |
| Symptomatic women,  n (%) | 55 (11.9) | 123 (75.5) | 0.00 | 22.87 (14.52-36.02) |
| Parity, mean ± SD | 1.23±0.45 | 1.42±0.57 | 0.00 | 2.07 (1.47-2.93) |
| Gestational age at delivery, mean ± SD, week | 38.74 ± 1.96 | 38.62 ± 1.84 | 0.48 | 0.97 (0.86-1.06) |
| Age at delivery |  |  |  |  |
| ≤29 | 254 (54.7) | 73 (44.8) | 0.01 | Reference |
| 30-34 | 167 (36.0) | 62 (38.0) | 0.20 | 1.29 (0.87-1.91) |
| ≥35 | 43 (9.3) | 28 (17.2) | 0.00 | 2.27 (1.32-3.90) |
| Prior vaginal deliveries, n, (%) | 22 (4.7) | 10 (6.1) | 0.49 | 1.31 (0.61-2.84) |
| Number of abortions  before CS |  |  |  |  |
| 0, n, (%) | 280 (60.3) | 92 (56.4) | 0.65 | Reference |
| 1-2, n, (%) | 158 (34.1) | 62 (38.0) | 0.36 | 1.19 (0.82-1.74) |
| ≥ 3, n, (%) | 26 (5.6) | 9 (5.5) | 0.90 | 1.05 (0.48-2.33) |
| **Operation^*^** |  |  |  |  |
| Twice CSs, n, (%) | 54 (11.6) | 45 (27.6) | 0.00 | 2.90 (1.86-4.52) |
| Bilateral tubal ligation, n, (%) | 29 (6.3) | 19 (11.7) | 0.03 | 1.98(1.08-3.64) |
| Emergency CS, n, (%) | 75 (16.2) | 50 (30.7) | 0.00 | 2.30 (1.52-3.47) |
| surgeon experience ≥ 10 y, n, (%) | 118 (25.4) | 31 (19.0) | 0.10 | 0.69 (0.44-1.07) |
| **Infection^*^** |  |  |  |  |
| Postpartum endometritis | 11(2.4) | 12(7.4) | 0.01 | 3.27 (1.42-7.57) |
| MSAF, n, (%) | 14 (3.0) | 20 (12.3) | 0.00 | 4.50 (2.21-9.13) |
| Cervical dilatation |  |  |  |  |
| 0 (cm) | 408 (87.9) | 121 (74.2) | 0.00 | Reference |
| 1-3 (cm), n, (%) | 38 (8.2) | 16 (9.8) | 0.27 | 1.42 (0.77-2.64) |
| 4-10 (cm), n, (%) | 18 (3.9) | 26 (16.0) | 0.00 | 4.87 (2.58-9.18) |
| PROM (≥ 24 h), n, (%) | 65 (14.0) | 42 (25.8) | 0.00 | 2.13 (1.38-3.30) |
| Vaginal examination, mean ± SD | 0.30 ± 0.61 | 0.50 ± 0.86 | 0.00 | 1.48 (1.16-1.88) |
| **Tension^*^** |  |  |  |  |
| Pre-pregnancy BMI, mean ± SD, kg/m^2^ | 22.13 ± 2.72 | 21.71 ± 2.67 | 0.09 | 0.94 (0.88-1.01) |
| BMI at delivery, mean ± SD, kg/m^2^ | 27.67 ± 3.13 | 27.68 ± 3.23 | 0.99 | 1.00 (0.95-1.06) |
| Retroflected uterus, n, (%) | 71 (15.3) | 104 (63.8) | 0.00 | 9.76 (6.49-14.66) |
| Macrosomia, n, (%) | 25 (5.4) | 7 (4.3) | 0.59 | 0.79 (0.33-1.86) |
| Twin pregnancy, n, (%) | 30 (6.5) | 11 (6.7) | 0.90 | 1.05 (0.51-2.14) |
| Breech, n, (%) | 34 (7.3) | 4 (2.5) | 0.03 | 0.32 (0.11-0.91) |
| Presence of labor before CS, n, (%) | 59 (12.7) | 42 (25.8) | 0.00 | 2.38 (1.53-3.72) |
| Duration of labor before CS, mean ± SD, h | 9.63±6.41 | 11.30±6.12 | 0.19 | 1.04 (0.98-1.11) |
| Oxytocin augmentation  during labor, n, (%) | 38 (8.2) | 25 (15.3) | 0.01 | 2.03 (1.18-3.49) |
| **Healing^*^** |  |  |  |  |
| Pre-eclampsia |  |  |  |  |
| No, n, (%) | 437(94.2) | 157(96.3) | 0.48 | Reference |
| Mild, n, (%) | 18(3.9) | 5(3.1) | 0.62 | 0.77(0.28-2.12) |
| Severe, n, (%) | 9(1.9) | 1(0.6) | 0.27 | 0.31(0.04-2.46) |
| Diabetes |  |  |  |  |
| No, n, (%) | 423(91.2) | 142(87.1) | 0.32 | Reference |
| Mild, n, (%) | 34(7.3) | 18(11.0) | 0.14 | 1.58(0.86-2.88) |
| Severe, n, (%) | 7(1.5) | 3(1.8) | 0.73 | 1.28(0.33-5.003) |
| ICP |  |  |  |  |
| No, n, (%) | 433(93.3) | 137(84.0) | 0.00 | Reference |
| Mild, n, (%) | 26(5.6) | 22(13.5) | 0.00 | 2.67(1.47-4.87) |
| Severe, n, (%) | 5(1.1) | 4(2.5) | 0.17 | 2.53(0.67-9.55) |
| Anemia, n, (%) | 78 (16.8) | 41 (25.2) | 0.02 | 1.66 (1.08-2.56) |
| PPH, mean ± SD, mL | 398.53 ± 168.85 | 439.79 ± 237.53 | 0.02 | 1.00 (1.00-1.00) |
| Placenta previa, n, (%) | 13 (2.8) | 4 (2.5) | 0.81 | 0.87 (0.28-2.72) |
| Steroid treatment during pregnancy, n, (%) | 80 (17.2) | 25 (15.3) | 0.58 | 0.87 (0.533-1.42) |
| ART, n, (%) | 44 (9.5) | 16(9.8) | 0.90 | 1.04 (0.57-1.90) |

MSAF: meconium-stained amniotic fluid; CS: cesarean section; ICP: intrahepatic cholestasis of pregnancy; PROM: premature rupture of membranes; BMI: bass mass index; PPH: postpartum hemorrhage; ART: assisted reproduction technology.

^*^ The variables were classified into four parts, including operation, infection, tension, and healing related risk factors.

Table S2 Demographic characteristics of participants including training and validation cohort

| **Basic information** |  |
| --- | --- |
| Large, n, (%) | 203 (27.1) |
| Age at delivery, mean ± SD, y | 29.87±3.84 |
| Gestational age at delivery, mean ± SD, week | 38.62±1.94 |
| Prior vaginal deliveries, n, (%) | 37 (4.9) |
| had one previous CS, n, (%) | 143 (19.1) |
| Number of abortions before CS | 307 (40.9) |
| Parity, mean ± SD | 1.14±0.59 |

CS: cesarean section, SD: standard deviation.

Reference

1. Mackeen AD, Packard RE, Ota E, Speer L. Antibiotic regimens for postpartum endometritis. Cochrane Database Syst Rev. 2015 Feb 2;2015(2):Cd001067.

2. Smaill FM, Grivell RM. Antibiotic prophylaxis versus no prophylaxis for preventing infection after cesarean section. Cochrane Database Syst Rev. 2014 Oct 28;2014(10):Cd007482.

3. Poon LC, Shennan A, Hyett JA, Kapur A, Hadar E, Divakar H, et al. The International Federation of Gynecology and Obstetrics (FIGO) initiative on pre-eclampsia: A pragmatic guide for first-trimester screening and prevention. Int J Gynaecol Obstet. 2019 May;145 Suppl 1(Suppl 1):1-33.

4. Lende M, Rijhsinghani A. Gestational Diabetes: Overview with Emphasis on Medical Management. Int J Environ Res Public Health. 2020 Dec 21;17(24).

5. Bicocca MJ, Sperling JD, Chauhan SP. Intrahepatic cholestasis of pregnancy: Review of six national and regional guidelines. Eur J Obstet Gynecol Reprod Biol. 2018 Dec;231:180-7.
